# Injury of the Mammillothalamic Tract in Patients with Thalamic Hemorrhage

**DOI:** 10.3389/fnhum.2014.00259

**Published:** 2014-04-22

**Authors:** Hyeok Gyu Kwon, Han Do Lee, Sung Ho Jang

**Affiliations:** ^1^Department of Physical Medicine and Rehabilitation, College of Medicine, Yeungnam University, Daegu, South Korea

**Keywords:** mammillothalamic tract, thalamic hemorrhage, diffusion tensor tractography, thalamus, papez circuit

## Abstract

**Objective:** Injury of the mammillothalamic tract (MTT) has been suggested as one of the plausible pathogenic mechanisms of memory impairment in patients with thalamic hemorrhage; however, it has not been clearly demonstrated so far. We attempted to investigate whether injury of the MTT documented by diffusion tensor tractography following thalamic hemorrhage correlates with cognitive impairment.

**Methods:** We recruited 22 patients with a thalamic hemorrhage and 20 control subjects. MTTs were reconstructed using the probabilistic tractography method. Patients were classified into two subgroups: reconstructed group, patients whose MTT was reconstructed in the affected hemisphere, and non-reconstructed group, patients whose MTT was not reconstructed.

**Results:** Mammillothalamic tract was reconstructed in 5 (22.7%, reconstructed group) patients in the affected hemisphere and was not reconstructed in the remaining 17 patients (77.3%, non-reconstructed group). In addition, the MTT was not reconstructed even in the unaffected hemisphere in four patients (23.5%) in non-reconstructed group. Fractional anisotropy and mean diffusivity values of the affected hemisphere in reconstructed group also did not show significant differences from those in the unaffected hemisphere of reconstructed group and the control group (*p* > 0.05). However, the tract volume of the affected hemisphere in reconstructed group was significantly lower than that of the unaffected hemisphere in reconstructed group and the control group (*p* < 0.05).

**Conclusion:** A large portion of patients with thalamic hemorrhage appeared to suffer severe injury of the ipsilesional MTT (77.3%) and 18.2% of them appeared to suffer severe injury even in the contralesional MTT. In addition, the remaining 22.7% of patients who had preserved integrity of the ipsilesional MTT appeared to suffer partial injury of the ipsilesional MTT.

## Introduction

The mammillothalamic tract (MTT), part of the Papez circuit, connects between the mammillary body and the anterior thalamus, and is involved in the episodic memory (Aggleton and Brown, 1999). Although the mammillary body, which is known to have the size of 63.5 mm^3^, and anterior thalamus are easily seen (Sheedy et al., 1999), the MTT has not easily discriminated from adjacent structures due to the anatomical characteristics of short and thin neural structure. Therefore, accurate isolation and evaluation of the MTT in the human brain have been difficult. Recent developments in diffusion tensor tractography (DTT), derived from diffusion tensor imaging (DTI), allow for three-dimensional reconstruction and localization of the MTT in the live human brain (Kwon et al., 2010).

Memory impairment is one of the various neurological manifestations in patients with thalamic hemorrhage (Choi et al., 1983; Kawahara et al., 1986; Waxman et al., 1986; Hankey and Stewart-Wynne, 1988; Kumral et al., 1995; Chen et al., 1996; Chung et al., 1996; Exner et al., 2001; Summers, 2002; Kuljic-Obradovic et al., 2007; Kalefa et al., 2008). Injury of the MTT has been suggested as one of the plausible pathogenic mechanisms of memory impairment in patients with thalamic lesions along with injury of the ventral amygdalofugal pathway and the thalamocortical pathway between the mediodorsal nucleus of the thalamus and the prefrontal cortex (Kawahara et al., 1986; Hankey and Stewart-Wynne, 1988; Graff-Radford et al., 1990; Chen et al., 1996; Exner et al., 2001; Hampstead and Koffler, 2009). However, it has not been clearly demonstrated so far.

In this study, we attempted to investigate whether injury of the MTT documented by DTT following thalamic hemorrhage correlates with cognitive impairment.

## Materials and Methods

### Subjects

We recruited 22 patients (male: 10, female: 12, mean age: 62.0 ± 9.3 years, range: 45–76 years) and 20 normal healthy control subjects (male: 10, female: 10, mean age: 54.2 ± 9.9 years, range: 42–71 years) with no previous history of neurological, physical, or psychiatric illness for this study. Inclusion criteria for patients were as follows:

(1) first ever stroke,
(2) a hematoma located primarily in the thalamus,
(3) DTI was scanned at early stage (between 1 and 5 weeks) after onset,
(4) no hydrocephalus, subarachnoid hemorrhage, intraventricular hemorrhage, or aphasia.

This study was conducted retrospectively and the study protocol was approved by the Institutional Review Board of our hospital.

### Clinical evaluation

Cognitive impairment in patients was evaluated at the time of DTI scanning. The Korean mini-mental state examination (K-MMSE) was used for the assessment of cognitive impairment. The reliability and validity of the K-MMSE have been well established (Dick et al., 1984; Han et al., 2008).

The location of hemorrhage was classified as follows: anterior, posteromedial, posterolateral, dorsal, and global type (Chung et al., 1996). Volume of hematoma was measured using ITK-SNAP program (University of Pennsylvania, Philadelphia, PA, USA) (Yushkevich et al., 2006).

### Diffusion tensor tractography

**Figure 1:**
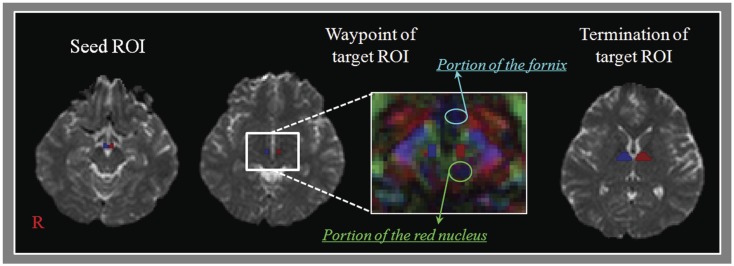
**Regions of interests (ROIs) for the mammillothalamic tract (MTT)**. Regions of interest – seed ROI is located at the mammillary body on the b0 map. The waypoint of target ROI is given at the MTT area (between the portion of the fornix and the red nucleus in the anteroposterior direction) at about the bicommissural level on the color map. The termination of target ROI is given at the portion of anterior thalamus on the b0 map.

Diffusion tensor imaging and MRI scanning were performed using a six-channel head coil on a 1.5-T Philips Gyroscan Intera (Philips Ltd., Best, The Netherlands) with single-shot echo-planar imaging (EPI). For each of the 32 non-collinear diffusion sensitizing gradients, we acquired 67 contiguous slices parallel to the anterior commissure–posterior commissure line. Imaging parameters of DTI were as follows: acquisition matrix = 96 × 96; reconstructed to matrix = 128 × 128; field of view = 221 mm × 221 mm; repetition time (TR) = 10,726 ms; echo time (TE) = 76 ms; parallel imaging reduction factor (SENSE factor) = 2; EPI factor = 49; *b* = 1000 s/mm^2^; number of excitations (NEX) = 1; and a slice thickness of 2.3 mm (acquired anisotropic voxel size 1.73 mm × 1.73 mm × 2.3 mm). Imaging parameters for T2-weighted MRI were as follows: acquisition matrix = 265 × 224, field of view = 210 mm × 210 mm, TR = 4224.1 ms, TE = 100 ms, NEX = 2, and slice thickness = 5 mm with a gap of 2.2 mm. Affine multi-scale two-dimensional registration at the Oxford Centre for Functional Magnetic Resonance Imaging of Brain (FMRIB) Software Library (FSL; www.fmrib.ox.ac.uk/fsl) was used for the removal of eddy current-induced image distortions (Smith et al., 2004). For skull strip, brain extraction tool in the FSL was used. Fiber tracking was performed using a probabilistic tractography method based on a multi-fiber model, and applied in this study utilizing tractography routines implemented in FMRIB Diffusion with BedpostX method (5000 streamline samples, 0.5 mm step lengths, curvature thresholds = 0.2) (Behrens et al., 2003, 2007; Smith et al., 2004). Regions of interests (ROIs) and fiber tracking were performed only in DTI. MTTs were determined by the selection of fibers passing through three ROIs (Figure 1). The seed ROI was located at the mammillary body, which is known anatomy on the axial image with b0 map. The waypoint of target ROI was given at the isolated MTT area (between the portion of the fornix and the red nucleus in the anteroposterior direction) at about the bicommissural level on the axial image with color map. The termination of target ROI was given at the portion of anterior thalamus on the axial image with b0 map (Duvernoy and Bourgouin, 1999; Kwon et al., 2010). In order to reconstruct for the integrity of the MTT between the mammillary body and the anterior thalamus, of 5000 samples generated from each seed voxel, results for each contact were the visualized threshold point at 1 streamline through each voxel for analysis. We measured values of fractional anisotropy (FA), mean diffusivity (MD), and tract volume of the MTT and size of three ROIs using MATLAB^TM^ (Matlab R2007b, The Mathworks, Natick, MA, USA). The mean size of ROIs for the mammillary body, waypoint, and anterior thalamus were 15.2 ± 1.6, 11.8 ± 2.2, and 67.0 ± 9.1 mm^2^, respectively. In addition, no significant difference was observed in the size of ROIs (seed, waypoint, and termination ROIs) between the reconstructed and non-reconstructed group.

For measurement of intra- and inter-observer, random analyses of the data were performed by two evaluators (Hyeok Gyu Kwon and Han Do Lee) who were blinded to the other evaluator’s data. The consistency rate of analyses with three tract turning angles by two evaluators were identical for 82 out of 84 hemispheres (97.6%), and two sets of analyses made by one analyzer (Hyeok Gyu Kwon) were identical for 84 out of 84 hemispheres (100%).

According to findings for the MTT on DTT by the preserved integrity between the mammillary body and the anterior thalamus, patients were classified into two subgroups: reconstructed group – patients whose MTT was reconstructed in the affected hemisphere and non-reconstructed group – patients whose MTT was not reconstructed in the affected hemisphere.

### Statistical analysis

SPSS software (v.15.0; SPSS Inc., Chicago, IL, USA) was used for data analysis. As for the control group, an independent *t*-test for determination of differences in the value of FA, MD, and tract volume was performed between hemispheres. Kruskal–Wallis test was used for determination of differences in FA, MD, and tract volume of the unaffected hemisphere between three groups and the Mann–Whitney *U*-test was used for determination of differences in FA, MD, and tract volume between the unaffected and the affected hemisphere in reconstructed group and between the affected hemisphere of reconstructed group and the control group and between the unaffected hemisphere of patient group (reconstructed group and non-reconstructed group, respectively) and the control group. Subsequently, we used the Mann–Whitney *U*-test for determination of differences in the K-MMSE and lesion volume between reconstructed group and non-reconstructed group. The significant level of the *p*-value was set at 0.05.

## Results

**Figure 2:**
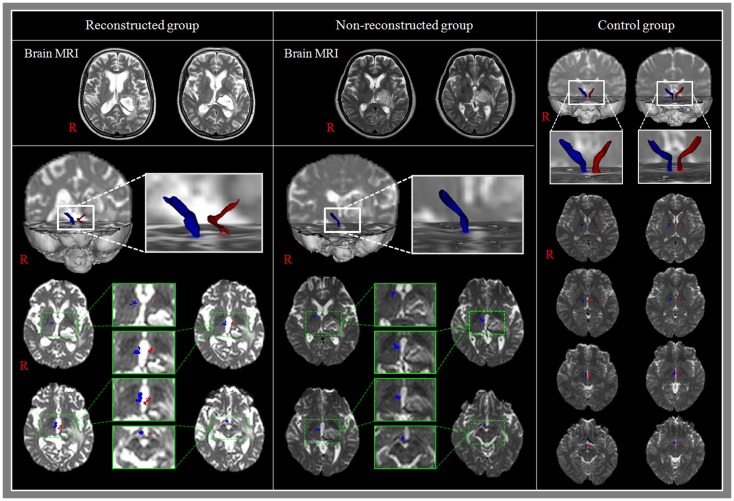
**Results of diffusion tensor tractography (DTT) for the mammillothalamic tract (MTT)**. Reconstructed group: T2-weighted brain MR images show the left thalamic hemorrhage and MTTs are reconstructed in both hemispheres. In addition, the MTT in the affected hemisphere is thin compared with that of the unaffected hemisphere. Non-reconstructed group: T2-weighted brain MR images show the left thalamic hemorrhage and the MTT in the affected hemisphere was not reconstructed.

Reconstructed MTTs showed the preserved integrity between the mammillary body and the anterior thalamus. In detail, MTTs which originated from the mammillary body, ascended posteriorly to the bicommissural level, along the third-ventricle, and then ascended to the anterior thalamus in the anterolateral direction (Figure 2). Among 22 patients, 5 patients belonged to reconstructed group [22.7%, male: 3, female: 2, mean age: 55.2 ± 7.7 years, lesion volume (mm^3^): 4397.6 ± 2733.2, K-MMSE: 25.0 ± 6.24] and the remaining 17 patients belonged to non-reconstructed group [77.3%, male: 7, female: 10, mean age: 64.0 ± 8.9 years, lesion volume (mm^3^): 5153.3 ± 2607.6, K-MMSE: 16.4 ± 9.28] (Table 1). However, the MTT was not reconstructed in the unaffected hemisphere in 4 (23.5%) of 17 patients in non-reconstructed group. Therefore, in measurement of the FA, MD, and tract volume of the MTT in the unaffected hemisphere, we excluded four patients of non-reconstructed group. With regard to the hematoma location, in reconstructed group, three patients belonged to the posterolateral type (13.6%) and the two remaining patients to the dorsal type (9.1%). By contrast, in non-reconstructed group, eight patients belonged to the posterolateral type (36.4%), eight patients to the global type (36.4%), and the remaining patient to the posteromedial type (4.6%).

**Table 1:** **Demographic and clinical data according to diffusion tensor tractography type for the patient and control groups**.

Variables	Reconstructed group	Non-reconstructed group	Control group
Sex (male:female)	3:2	7:10	10:10
Mean age (years)	55.2 (7.7)	64.0 (8.9)	54.2 (9.9)
Lesion side (Rt:Lt)	2:3	8:9	
Lesion volume (mm^3^)	4397.6 (2733.2)	5153.3 (2607.6)	
Hemorrhage type (Ant:PL:PM:Dor:Glo)	0:3:0:2:0	0:8:1:0:8	
Mean days to DTT or duration from onset (days)	20.2 (10.9)	18.5 (8.5)	
Risk factor (smoking:alcohol:HTN:DM)	1:1:1:0	3:2:8:1	
K-MMSE	25.0 (6.24)	16.4 (9.28)	

*Values represent mean (±SD), Ant, anterior type; PL, posterolateral type; PM, posteromedial type; Dor, dorsal type; Glo, global type; DTT, diffusion tensor tractography; HTN, hypertension; DM, diabetes mellitus; K-MMSE, Korean mini-mental state examination*.

A summary of the results of DTT parameters for the MTT in the patient and control groups is shown in Table 2. Regarding the FA, MD, and tract volume, we did not observe significant differences between the unaffected hemisphere of the patient group (reconstructed group and non-reconstructed group, respectively) and control groups (*p* > 0.05). Likewise, no difference was observed between the unaffected hemisphere of reconstructed group and non-reconstructed group in the patient group and between the right- and left-hemisphere in control group (*p* > 0.05). FA and MD values of the affected hemisphere in reconstructed group also did not show significant differences from those in the unaffected hemisphere of reconstructed group and the control group (*p* > 0.05). However, the tract volume of the affected hemisphere in reconstructed group was significantly lower than that of the unaffected hemisphere in reconstructed group and the control group (*p* < 0.05). As for the K-MMSE, we observed significant difference between reconstructed group and non-reconstructed group (*p* < 0.05). However, no significant difference in lesion volume was observed between reconstructed group and non-reconstructed group (*p* > 0.05).

**Table 2:** **Diffusion tensor tractography parameters of the mammillothalamic tract in the patient groups and control group**.

	Unaffected hemisphere			Affected hemisphere		
	FA	MD	Tract volume	FA	MD	Tract volume
Reconstructed group	0.36 (0.04)	0.94 (0.11)	67.60 (18.38)	0.34 (0.04)	0.97 (0.10)	32.60^a^ (9.74)
Non-reconstructed group	0.36 (0.05)	0.96 (0.13)	73.86 (24.78)			

	**Right**			**Left**		
	**FA**	**MD**	**Tract volume**	**FA**	**MD**	**Tract volume**

Control group	0.37 (0.04)	0.89 (0.11)	70.45 (16.96)	0.38 (0.11)	0.90 (0.06)	74.75 (18.87)

*Values represent mean (±SD), FA, fractional anisotropy; MD, mean diffusivity MD × 10^−3^ (mm^2^/s)*.

*Unit of tract volume: voxel*.

*^a^Significant differences between unaffected and affected hemisphere in Reconstructed group and between affected in the Reconstructed group and both hemispheres in control group*.

## Discussion

In this study, we investigated injury of the MTT in patients with thalamic hemorrhage using DTT. We classified the patients into two groups according to preservation of integrity of the MTT in the affected hemisphere. According to our findings, the MTT was reconstructed in 22.7% of patients in the affected hemisphere and was not reconstructed in the remaining patients (77.3%). In addition, the MTT was not reconstructed even in the unaffected hemisphere in 23.5% of patients in non-reconstructed group, which corresponded with 18.2% of a total of 22 patients. These results suggest that a large portion of patients with thalamic hemorrhage appeared to suffer severe injury of the contralesional MTT (18.2%) as well as the ipsilesional MTT (77.3%).

The tract volume of the affected hemisphere in reconstructed group was decreased compared with those of the unaffected hemisphere in reconstructed group and the control group without significant change of FA and MD values. FA value represents the degree of directionality of microstructures, such as axons, myelin, and microtubules, and MD value indicates the magnitude of water diffusion (Mori et al., 1999; Assaf and Pasternak, 2008; Neil, 2008). By contrast, the tract volume is determined by the total number of voxel in a neural tract (Kwak et al., 2010). Therefore, these results showing the decrement of tract volume of the MTT in the affected hemisphere without change of FA and MD values suggest decreased fiber number of the MTT. It appears to indicate partial injury of the MTT even though in patients who had preserved integrity of the MTT in the affected hemisphere. On the other hand, these changes of DTI parameters may also be attributed to the local effects such as perilesional compression or edema without disintegration of neural fibers of the MTT.

With regard to the K-MMSE, we observed significant difference between reconstructed group and non-reconstructed group, indicating that preservation of integrity of the MTT in the affected hemisphere affected the whole cognition. On the other hand, no significant difference in lesion volume was observed between reconstructed group and non-reconstructed group. This result suggests that lesion volume did not affect the presence of injury severity of the MTT. However, we found that the global-type hematoma was prevalent in non-reconstructed group (8 of 17 patients) and dorsal type was prevalent in reconstructed group (2 of 5 patients). As a result, it appears that the proximity of the hematoma to the MTT is an important determinant of occurrence of MTT injury irrespective of the hematoma volume. However, due to the small number of patients, we could not determine injury of the MTT according to hematoma location in the thalamus. Therefore, further studies on this topic involving large numbers of patients will be necessary.

Many previous studies have reported memory impairment in thalamic hemorrhage, although the exact prevalence of memory impairment in patients with thalamic hemorrhage has not been reported (Choi et al., 1983; Kawahara et al., 1986; Waxman et al., 1986; Hankey and Stewart-Wynne, 1988; Kumral et al., 1995; Chen et al., 1996; Chung et al., 1996; Exner et al., 2001; Summers, 2002; Kuljic-Obradovic et al., 2007; Kalefa et al., 2008). Some of these studies have reported correlation of memory impairment with the location of hematoma, in detail, posteromedial type or posterolateral type (Waxman et al., 1986; Kumral et al., 1995; Chung et al., 1996). In 1996, Chung et al. investigated the clinical courses and outcome according to the type of location of hemorrhage in 157 patients with thalamic hemorrhage, and they found that the incidence of thalamic sub-type (anterior, posteromedial, posterolateral, dorsal, and global) were 6, 14, 44, 18, and 18%, respectively. In addition, it was found that the patients with posteromedial type showed severe memory impairment (Chung et al., 1996). On the other hand, several studies have reported on cognitive impairment in patients who have pathology in the MTT, including thalamic infarct, Korsakoff syndrome, and Wernicke’s encephalopathy (Schott et al., 2003; Yoneoka et al., 2004; Park et al., 2007; Kim et al., 2009, 2010). However, because these studies were conducted using conventional brain MRI or resting-state functional MRI, they have not directly demonstrated injury of the MTT.

In conclusion, a large portion of patients with thalamic hemorrhage appeared to suffer severe injury of the ipsilesional MTT (78.3%) and 17.4% of them appeared to suffer severe injury even in the contralesional MTT. In addition, the remaining 21.7% of patients who had preserved integrity of the ipsilesional MTT appeared to suffer partial injury of the ipsilesional MTT. These results suggest that patients with thalamic hemorrhage are at high risk of MTT injury, and that these patients should be thoroughly evaluated for MTT injury. To the best of our knowledge, this is the first DTI study of MTT injury in stroke patients. The major limitation of this study is the lack of direct detailed clinical data regarding the function of MTT, which could not be included due to the retrospective nature of the study. In addition, patients with a large hemorrhage may move more in the scanner causing artifact, which would affect our results. Another limitation was the small number of patients. Therefore, conduct of further prospective studies that include memory function and a larger number of patients should be encouraged. On the other hand, limitations of DTI should be considered. In particular, DTI may underestimate fiber tracts due to hematoma or peri-lesional edema in patients with thalamic hemorrhage (Lee et al., 2005; Parker and Alexander, 2005; Yamada et al., 2009).

## Conflict of Interest Statement

The authors declare that the research was conducted in the absence of any commercial or financial relationships that could be construed as a potential conflict of interest.
